# Traumatic brain injury, antisocial personality disorder and alcohol

**DOI:** 10.1192/j.eurpsy.2022.1714

**Published:** 2022-09-01

**Authors:** C. De Andrés Lobo, T. Jiménez Aparicio, C. Vallecillo Adame, M. Queipo De Llano De La Viuda, A. Gonzaga Ramírez, G. Guerra Valera, I. Santos Carrasco, J. Gonçalves Cerejeira, M. Fernández Lozano, B. Rodríguez Rodríguez, N. Navarro Barriga, M.J. Mateos Sexmero, N. De Uribe Viloria, G. Medina Ojeda

**Affiliations:** 1 Hospital Clínico Universitario, Psiquiatría, Valladolid, Spain; 2 Hospital Clínico Universitario de Valladolid, Psychiatry, Valladolid, Spain; 3 Hospital Universitario Fundación de Alcorcón, Psiquiatría, Alcorcón, Spain; 4 Sacyl, Hospital Clínico Universitario Valladolid, Psiquiatría, Valladolid, Spain

**Keywords:** traumatic brain injury, alcohol, antisocial behavior

## Abstract

**Introduction:**

Traumatic brain injury (TBI) can cause changes in the personality and behaviors. History of TBI has been associated with violent behavior and substance abuse.

**Objectives:**

Presentation of a clinical case of a patient with antisocial personality traits who suffered a TBI and abuses alcohol.

**Methods:**

We conducted a bibliographic review by searching for articles published the last 5 years in Pubmed

**Results:**

We present the case of a 48-year-old male patient with a history of myoclonic epilepsy, who suffered a TBI in a car crash. During his stay at ICU antisocial and borderline personality traits were found. When he came to consultations, he presented signs of alcohol intoxication (verbiage with hasty and dysarthric speech, and psychomotor incoordination). He acknowledges daily alcohol intake, although he minimizes it. During the interview he is irritable, prone to anger when contradicted and boasts of episodes of heteroaggressiveness and violence that he has carried out in the past. He reports morning sickness and tremors, but does not accept that they may be due to alcohol withdrawal. There is no motivation for change.

**Conclusions:**

It has been determined that history of TBI is more frequent in individuals with antisocial personality. TBI has been linked to violent behaviors, poor inhibitory control, engaging in illegal acts and higher rates of substance abuse. However, the causal relationship between antisocial behavior and TBI has yet to be clarified, as the available evidence does not show which comes first. More research is needed in the future that takes into account the temporal sequence of events.

**Disclosure:**

No significant relationships.

